# Persistent neuropathological effects 14 years following amyloid-β immunization in Alzheimer’s disease

**DOI:** 10.1093/brain/awz142

**Published:** 2019-06-03

**Authors:** James A R Nicoll, George R Buckland, Charlotte H Harrison, Anton Page, Scott Harris, Seth Love, James W Neal, Clive Holmes, Delphine Boche

**Affiliations:** 1 Clinical Neurosciences, Clinical and Experimental Sciences, University of Southampton, Southampton, UK; 2 Department of Cellular Pathology, University Hospital Southampton NHS Foundation Trust, Southampton, UK; 3 Biomedical Imaging Unit, Faculty of Medicine, University of Southampton, Southampton, UK; 4 Public Health Sciences and Medical Statistics, Faculty of Medicine, University of Southampton, Southampton, UK; 5 Department of Neuropathology, Institute of Clinical Neurosciences, School of Clinical Sciences, University of Bristol, Bristol, UK; 6 Department of Cellular Pathology, University Hospital of Wales, Cardiff University, Cardiff, UK; 7 Memory Assessment and Research Centre, Moorgreen Hospital, Southern Health Foundation Trust, Southampton, UK

**Keywords:** Alzheimer’s disease, dementia, immunotherapy, neuropathology, amyloid-β

## Abstract

We performed a 15-year post-mortem neuropathological follow-up of patients in the first trial of amyloid-β immunotherapy for Alzheimer’s disease. Twenty-two participants of a clinical trial of active amyloid-β_42_ immunization (AN1792, Elan Pharmaceuticals) or placebo were studied. Comprehensive post-mortem neuropathological assessments were performed from 4 months to 15 years after the trial. We analysed the relationships between the topographical distribution of amyloid-β removal from the cerebral cortex and tau pathology, cerebrovascular territories, plasma anti-AN1792 antibody titres and late cognitive status. Seventeen of 22 (77%) participants had Alzheimer’s neuropathological change, whereas 5 of 22 (23%) had alternative causes for dementia (progressive supranuclear palsy = 1, Lewy body disease = 1, vascular brain injury = 1, and frontotemporal lobar degeneration = 2). Nineteen of the 22 participants had received the active agent, three the placebo. Fourteen of 16 (88%) patients with Alzheimer’s disease receiving the active agent had evidence of plaque removal (very extensive removal = 5, intermediate = 4, very limited = 5, no removal = 2). Of particular note, two Alzheimer’s patients who died 14 years after immunization had only very sparse or no detectable plaques in all regions examined. There was a significant inverse correlation between post-vaccination peripheral blood anti-AN1792 antibody titres and post-mortem plaque scores (ρ* =* − 0.664, *P = *0.005). Cortical foci cleared of plaques contained less tau than did cortex with remaining plaques, but the overall distribution of tangles was extensive (Braak V/VI). In conclusion, patients with Alzheimer’s disease actively immunized against amyloid-β can remain virtually plaque-free for 14 years. The extent of plaque removal is related to the immune response. This long duration of efficacy is important in support of active immunization protocols as therapy for, or potentially prevention of, neurodegeneration-associated protein accumulations. Inclusion of patients without Alzheimer’s disease in Alzheimer’s therapy trials is a problem for assessing the efficacy of treatment. Despite modification of Alzheimer’s pathology, most patients had progressed to severe dementia, notably including the five with very extensive plaque removal, possibly due to continued tau propagation. Neuropathology follow-up of patients in therapeutic trials provides valuable information on the causes of dementia and effects of treatment.

## Teaser

The first human clinical trial of Aβ immunisation in Alzheimer’s disease began almost 20 years ago. In a postmortem follow-up study, Nicoll *et al.* reveal persistent Aβ plaque removal up to 14 years after immunisation. Tau continued to spread, however, possibly explaining why most patients progressed to severe dementia.

See Overk and Masliah (doi:10.1093/brain/awz165) for a scientific commentary on this article.

## Introduction

The concept of immunization against amyloid-β for the treatment of Alzheimer’s disease has been under investigation for nearly 20 years. The amyloid cascade hypothesis states that amyloid-β abnormalities play a key initiating role in Alzheimer’s disease pathophysiology, making amyloid-β a target for the treatment or prevention of the disease (Selkoe and Hardy, 2016). In a mouse model, amyloid-β immunization early in life prevented amyloid-β plaque formation (Schenk *et al.*, 1999), whereas in aged mice it reduced the plaque burden (Schenk *et al.*, 1999) and protected against neurological dysfunction (Morgan *et al.*, 2000).

The same experimental approach, using multiple peripheral inoculations of full-length amyloid-β_42_ peptide with adjuvant, designated AN1792 (Elan Pharmaceuticals), resulted in a human clinical trial that began in 2000 (Bayer *et al.*, 2005). The first post-mortem neuropathological study of a patient receiving amyloid-β immunization (Nicoll *et al.*, 2003) suggested profound changes in Alzheimer’s pathology, prompting us to instigate a systematic clinical and neuropathological follow-up of participants in the AN1792 trial. The main aims of that initial clinical trial were to evaluate the safety and immunogenicity of AN1792 (Bayer *et al.*, 2005). Eighty patients were recruited with a clinical diagnosis of mild to moderate Alzheimer’s disease. Sixty-four patients received the active agent with variable doses of AN1792 (50 or 225 µg) and variable doses of adjuvant (QS-21, 50 or 100 µg) and 16 patients received adjuvant alone. Patients received four injections over a 24-week period and some received up to four further injections of a modified formulation up to Week 72 of the study. During the period of the first four injections, 23.4% of patients developed detectable circulating anti-AN1792 antibodies, rising to 58.8% of patients after additional injections. Although the trial was not designed to assess efficacy, one of four clinical assessments used (Disability Assessment for Dementia) showed significantly less decline in the treated group than the controls after 84 weeks (Bayer *et al.*, 2005). Our clinical follow-up at 6 years showed no evidence of improved survival or delayed development of severe dementia in treated patients compared with those receiving the placebo (Holmes *et al.*, 2008). Even very extensive removal of amyloid-β plaques from the brain seemed insufficient to halt cognitive decline, although this conclusion was based on only two patients (Holmes *et al.*, 2008) and was thought to require confirmation in a larger sample.

Consequently, here we report a 15-year post-mortem neuropathology follow-up study of 22 of the 80 participants in the first clinical trial of amyloid-β immunotherapy (Bayer *et al.*, 2005). Our aims were: (i) to perform neuropathological assessment to determine the accuracy of the clinical diagnosis of Alzheimer’s disease in the setting of this trial; (ii) to assess the duration of effects of immunotherapy on neuropathology and dementia status, many years after treatment; (iii) to explore factors that might contribute to the variability of responses, in particular the relationship of the extent of amyloid-β removal to circulating anti-AN1792 antibody titres and to cerebrovascular territories; and (iv) to analyse the effects of amyloid-β removal on tau pathology in this unique cohort of immunized Alzheimer’s cases.

## Patients and methods

### Subjects, ethics and consent

Ethical approval was obtained to follow up participants in the trial and seek their consent for post-mortem brain donation (Southampton and South West Hampshire Local Research Ethics Committees, Reference No. LRC 075/03/w). Twenty-two participants and their carers consented and brain donations were complete by the end of 2015. Trial data were made available by Elan Pharmaceuticals and included: duration of dementia at trial entry, AN1792 dose, AN1792 number of doses, assays of peripheral blood anti-AN1792 antibodies, *APOE* genotypes and date of first immunization dose (Table 1). Subsequent assessment of dementia at a late stage prior to death was based on the last known assessment of activities of daily living scores and cognitive scores. Causes of death were obtained from death certificates.

**Table 1:** Clinical characteristics of subjects in the AN1792 trial who had post-mortem neuropathology

**Case ID**	**AN1792 dose**, **µg**	**Number of injections**	**Anti-AN1792 antibody response, mean titre**	**Survival time from first immunization, months**	**Duration of dementia, years**	**Last known dementia status**	**Age at death, years**	**Clinical cause of death (death certificate)**	***APOE* ɛ4 allele copy number**
**Neuropathologically confirmed Alzheimer’s disease**									
1	50	5	119	20	6	Severe	74	Pulmonary embolism, DVT, neurological disorder	1
2	50	3	0	4	11	Moderate	83	Rupture of abdominal aortic aneurysm, coronary artery atherosclerosis	0
3	225	4	0	41	6	Severe	63	Chronic neurodegenerative disease, epilepsy	0
4	225	8	4072	44	10	Severe	71	Alzheimer’s disease	0
6	50	8	1707	57	7	Severe	81	Old age, Alzheimer’s disease	1
7	50	8	4374	60	6	Severe	82	Old age, Alzheimer’s disease, cerebral atrophy	1
8	50	8	6470	64	10	Severe	63	Ischaemic heart disease, coronary artery atherosclerosis	1
9	225	7	491	63	11	Severe	81	Ischaemic heart disease, coronary artery atherosclerosis	2
10	50	7	137	86	11	Moderate	88	Bronchopneumonia, dementia	0
11	50	8	142	94	12	Moderate	88	Bronchopneumonia and mucinous cystadenoma of pancreas (stented), dementia	1
15	Placebo	n/k	7	104	12	Severe	93	Heart failure, ischaemic heart disease, diabetes mellitus, Alzheimer’s disease	1
16	225	6	142	111	15	Severe	89	Cerebrovascular accident, Dementia	1
17	50	n/k	0	141	13	Severe	86	Bronchopneumonia, Alzheimer’s disease	2
19	50	8	221	162	19	Severe	75	Dementia	n/a
20	225	3	430	166	17	Severe	82	Alzheimer’s disease	n/a
21	50	8	3045	173	18	Severe	87	Lower respiratory tract infection, dementia	1
22	225	n/k	1313	184	18	Severe	74	Alzheimer’s disease	2
**Non-Alzheimer’s disease**									
5	50	8	0	51	6	Severe	79	Aspiration pneumonia, Alzheimer’s disease and cerebrovascular disease, lung cancer	0
12	225	n/k	5455	89	9	Severe	86	Alzheimer’s disease	n/a
13	225	n/k	0	90	11	Severe	90	Pneumonia, CVA, dementia	0
14	Placebo	8	0	96	9	Severe	64	Alzheimer’s disease	2
18	Placebo	n/k	0	153	16	Severe	70	Alzheimer’s disease	0

CVA = cerebrovascular accident; DVT = deep vein thrombosis; n/a = not available; n/k = not known.

### Neuropathological diagnoses

All assessments were performed blind to the treatment status of the cases. Brains were examined and sampled for histology by standard methods after fixation in formalin. From each case, additional large coronal slices of cerebral hemisphere were processed. Paraffin sections were stained with haematoxylin and eosin, and immunohistochemistry was performed with antibodies including pan-amyloid-β (clone 6F/3D, Novocastra), tau (phosphorylated and non-phosphorylated tau, clone Tau-2, Sigma), phosphorylated (p)tau (clone AT8 Autogen Bioclear) and CD3 (pan-T lymphocytes, Abcam). Additionally, antibodies to α-synuclein (clone KM51, Novocastra), ubiquitin (clone ab7780, Abcam) and TDP43 (clone 10782–2-AP, Proteintech) were used when required for diagnosis. National Institute on Aging-Alzheimer’s Association guidelines for the neuropathological assessment of Alzheimer’s disease were used (Hyman *et al.*, 2012; Montine *et al.*, 2012).

### Amyloid-β assessments

#### Method 1

Sections immunolabelled for amyloid-β from all cerebral cortical regions sampled, including frontal, temporal, parietal and occipital lobes, representing both hemispheres, were assessed for histological features associated with immunization-mediated plaque removal, as defined previously: residual plaque cores in plaque-free areas, moth-eaten appearance of remaining plaques, phagocytosed amyloid-β within microglia, and marked cerebral arteriolar and capillary amyloid angiopathy (Nicoll *et al.*, 2006). Plaque removal was scored semi-quantitatively as: very extensive (i.e. nearly complete clearance of plaques, +++), intermediate (i.e. multiple and/or extensive plaque-free foci each involving a >1 cm length of cortical ribbon, ++), very limited (i.e. single and/or small plaque-free foci each involving a <1 cm length of cortical ribbon, +) or no evidence of plaque removal (−).

#### Method 2

Large coronal paraffin sections of a single cerebral hemisphere from each case were immunolabelled for amyloid-β and digitally scanned, the cortex was overlaid with 4 mm^2^ squares tiled contiguously such that the entire neocortical ribbon was sampled, and each box was scored for plaques in a manner adapted from the Consortium to Establish a Registry for Alzheimer’s disease (CERAD) protocol (frequent = 3, moderate = 2, sparse = 1, none = 0) (Mirra *et al.*, 1991). The results were expressed as amyloid-β plaque score, indicating the mean of the score in all boxes per subject, and as the percentage of boxes for each category represented graphically by a colour code (frequent = red, moderate = yellow, sparse = green, none = blue).

Cerebrovascular territories (anterior, middle and posterior cerebral arteries) (Kalaria *et al.*, 2015) were delineated on the scanned images. Watershed zones, defined as 20 squares each of 4 mm^2^ spanning the arterial territory boundaries, were compared with the remaining squares remote from the watershed zones.

Peak anti-AN1792 antibody titres were obtained from the original study data provided by Elan Pharmaceuticals (Bayer *et al.*, 2005). Mean titres were calculated, defined as total serum anti-AN1792 antibody titre in ELISA units over the period of treatment for each participant divided by the number of assays for that individual (Bayer *et al.*, 2005; Holmes *et al.*, 2008).

### Tau assessment

In neuropathologically confirmed Alzheimer’s cases, areas of neocortex were classified as either containing amyloid-β plaques (designated amyloid-β+) or lacking amyloid-β plaques (designated amyloid-β−), corresponding to the areas from which plaques had been extensively removed (Method 1). Corresponding areas of neocortex were identified in parallel sections immunolabelled for tau, images were captured from 10 microscope fields (×20 objective) and assessed for: tau-immunoreactive neuronal cell bodies (expressed as *n*/10 fields); dystrophic neurite clusters (expressed as *n*/10 fields) and neuropil thread density (designated sparse, moderate or frequent and expressed/field) (Alafuzoff *et al.*, 2008).

### Statistical analysis

Assessment of normality of the variables amyloid-β plaque score, mean and peak anti-AN1792 antibody response, and each tau feature was determined by examination of quantile-quantile plots. The relationship of amyloid-β plaque score to the immune response (mean and peak anti-AN1792 antibody titres) was investigated by non-parametric Spearman rank correlation. To assess whether the survival time related to the extent of plaque removal, a parametric independent samples t-test was used to compare survival in the five subjects with the most extensive plaque clearance with that in the remaining Alzheimer’s subjects. To explore the effect of amyloid-β plaque removal on tau pathology, the number of tangles and dystrophic neurites, and the neuropil thread density were compared between regions with or without amyloid-β plaques, by non-parametric Mann-Whitney U-test. Continuously distributed data are displayed using box-and-whisker plots, while the scoring expressed as percentage is illustrated using pie charts. To assess whether amyloid-β plaque score was linked to the vascular supply, one-way ANOVA was performed to assess difference between the cerebrovascular regions, and independent samples *t*-test between the watershed and non-watershed areas. SPSS software (version 24) was used for all analyses and *P*-values <0.05 were considered statistically significant throughout.

### Data availability

The data used and/or analysed during the current study are available from the corresponding author on reasonable request.

## Results

### Clinical details of participants

Clinical details of AN1792 trial participants included in the post-mortem neuropathological follow-up study are shown in Table 1. The duration of dementia from time of diagnosis to death ranged from 6 to 19 years. All patients had a clinical diagnosis of probable mild to moderate Alzheimer’s disease (14–26/30 points on the MMSE) (Bayer *et al.*, 2005; Holmes *et al.*, 2008). Overall, 19/22 participants had received the active agent (AN1792) and three the adjuvant alone as a placebo. Fourteen of the 19 patients (74%) had developed detectable antibodies to AN1792 in peripheral blood. Survival times after the first immunization varied widely, from 4 to 184 months. The clinical side-effect associated with amyloid-β immunotherapy with AN1792, termed ‘meningoencephalitis’ (Orgogozo *et al.*, 2003), was observed in only 1 of 22 patients (Case 1) (Nicoll *et al.*, 2003) (Table 2). *APOE* genotypes were typical of a population with dementia: 11/14 Alzheimer’s cases and one of the four non-Alzheimer’s cases with known *APOE* genotypes were *APOE* ɛ4 carriers.

**Table 2:** Neuropathological findings of subjects in the AN1792 trial

Case ID	Braak stages	Treatment status	Neuropath diagnosis	Histological evidence of plaque removal Method 1	Amyloid-β plaque score Method 2	Meningoencephalitis	AD neuropathological change	Additional findings
**Neuropathologically confirmed Alzheimer’s disease**								
1	V/VI	Immunized	AD	++	0.7	Yes	A3, B3, C3	Numerous cortical microvascular lesions related to severe CAA/ meningoencephalitis
2	III/IV	Immunized	AD	++	1.8	No	A3, B2, C2	Severe CAA, widespread capCAA
3	V/VI	Immunized	AD	−	2.1	No	A3, B3, C3	Focally severe CAA
4	V/VI	Immunized	AD	+	1.5	No	A3, B3, C3	Severe CAA
6	V/VI	Immunized	AD	++	1.6	No	A3, B3, C3	Severe CAA
7	V/VI	Immunized	AD	+++	0.05	No	A1, B3, C0	−
8	V/VI	Immunized	AD	+++	0.5	No	A1, B3, C1	−
9	V/VI	Immunized	AD/LBD	++	0.6	No	A3, B3, C1	Neocortical Lewy bodies, severe CAA
10	V/VI	Immunized	AD	+	2.9	No	A3, B3, C3	−
11	V/VI	Immunized	AD	+	2.3	No	A3, B3, C2	Severe CAA
15	V/VI	Placebo	AD/VBI	−	2.4	No	A3, B3, C3	Severe arteriolosclerosis/rarefaction of cerebral white matter, several cortical microinfarcts, moderate CAA
16	V/VI	Immunized	AD	+++	0.4	No	A3, B3, C0	Extensive capCAA, small acute infarct left basal ganglia
17	V/VI	Immunized	AD	+	2.7	No	A3, B3, C3	Small old right parietal infarct, microvascular lesions entorhinal cortex
19	V/VI	Immunized	AD	−	2.1	No	A3, B3, C3	Severe CAA
20	V/VI	Immunized	AD	+++	1	No	A3, B3, C2	Extensive capCAA
21	V/VI	Immunized	AD	+++	0.6	No	A3, B3, C1	Extensive capCAA
22	V/VI	Immunized	AD	+	2.3	No	A3, B3, C3	Extensive capCAA
**Non-Alzheimer’s disease**								
5	n/a	Immunized	PSP	−	0.1	No	A1, B(n\a), C0	Tau (neurons, microglia, astrocytes) brainstem, basal ganglia, neocortex
12	III/IV	Immunized	LBD, diffuse	^∗^	0.1	No	A2, B2, C0	Neocortical and nigral Lewy bodies, widespread capCAA
13	III/IV	Immunized	VBI	−	0.02	No	A0, B2, C0	Extensive temporal cortical infarction, basal ganglia infarction
14	I/II	Placebo	FTLD-TDP	−	1.5	No	A3, B1, C0	TDP43+ inclusions, hippocampal sclerosis
18	I/II	Placebo	FTLD-TDP	−	0.3	No	A1, B1, C0	TDP43+ inclusions

AD = Alzheimer’s disease; capCAA = capillary angiopathy; CVA = cerebrovascular accident, CAA = cerebral amyloid angiopathy; DVT = deep vein thrombosis; FTLD-TDP = frontotemporal lobar degeneration-TDP43; LBD = Lewy body disease, neocortical (diffuse); n/a = not applicable; PSP = progressive supranuclear palsy; VBI = vascular brain injury.

Method 1: Analysis of plaque removal was scored semi-quantitatively in frontal, temporal, parietal and occipital neocortex as: very extensive (i.e. nearly complete clearance of plaques) = +++; intermediate (i.e. multiple and/or extensive plaque-free foci each involving a >1 cm length of cortical ribbon) = ++; very limited (i.e. single and/or small plaque-free foci each involving each involving a <1 cm length of cortical ribbon) = +; no evidence of plaque removal = −.

Method 2: Quantification of amyloid-β plaques in coronal hemisphere sections (score out of 3).

^∗^LBD with marked capillary angiopathy but no plaques, possibly reflecting clearance of diffuse plaques.

Alzheimer's disease neuropathological change: A = amyloid-β plaque score; B = neurofibrillary tangle stage; C = neuritic plaque score according to National Institute on Aging-Alzheimer’s Association guidelines (Hyman *et al.*, 2012).

### Neuropathological diagnoses

The neuropathological analyses are shown in Table 2. Seventeen of the 22 cases (77%) had a distribution of tangles and at least some residual amyloid-β pathology indicating the cause of dementia had been Alzheimer’s disease (Table 2), of which 16 were Braak tangle stage V/VI (B3) and one case, a patient who had died just 4 months after the first immunization, was Braak stage III/IV (B2). Of the 17 neuropathologically confirmed Alzheimer’s cases, 16 had received the active agent and one the placebo. Of the five non-Alzheimer’s cases, three had received the active agent and two the placebo. Five of the 22 participants (23%) had non-Alzheimer’s causes for dementia: progressive supranuclear palsy (*n = *1), Lewy body disease (*n = *1), vascular brain injury (*n = *1) and frontotemporal lobar degeneration (FTLD-TDP43, *n = *2) (Table 2).

### Evidence of plaque clearance

#### Method 1

**Figure 1:**
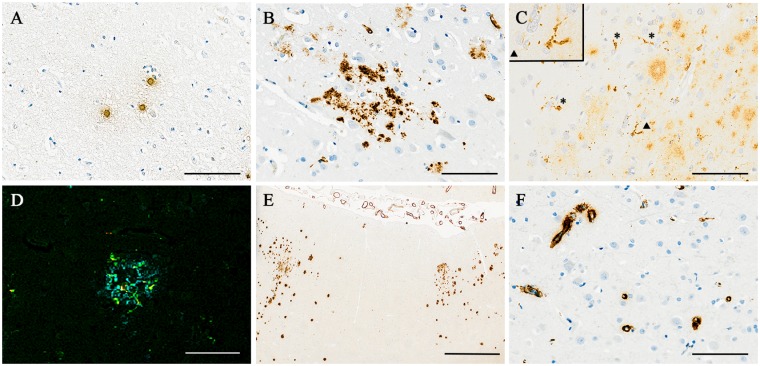
**Illustrations of the morphological features of amyloid-β immunohistochemistry associated with plaque clearance.** (**A**) Plaque-free areas of cerebral cortex containing scattered ‘naked’ residual plaque cores, lacking surrounding diffuse amyloid-β. (**B**) A ‘moth-eaten’ appearance of some remaining plaques. (**C**) A granular pattern of intracytoplasmic amyloid-β within microglia (arrows) with some ‘moth-eaten’ extracellular amyloid-β (*right* of image). (**D**) Triple label confocal image demonstrating microglia clustered around a remaining plaque (amyloid-β, cyan; HLA-DR, green; CD68, red; overlap, yellow). (**E**) A small cortical area lacking plaques, with remaining plaques on either side, associated with full-thickness, full-circumference amyloid-β staining of overlying leptomeningeal arteries indicating severe cerebral amyloid angiopathy. (**F**) Numerous capillaries with amyloid-β staining, indicating capillary angiopathy, in a plaque-free area of cerebral cortex. (**A**–**C**, **E** and **F**) Immunohistochemistry for pan-amyloid-β. (**D**) Triple label confocal microscopy for amyloid-β, HLA-DR and CD68. Scale bars = 100 μm in **A**–**C** and **F**; 50 μm in **D**; 1 mm in **E**.

Amyloid-β-immunolabelled sections from all cerebral cortical regions sampled were assessed for the specific histological features we previously associated with immunization-mediated plaque removal in Alzheimer’s disease (Table 2) (Nicoll *et al.*, 2006; Holmes *et al.*, 2008). These morphological markers of plaque clearance, also validated by their occurrence in PDAPP transgenic mice immunized with AN1792 (Schenk *et al.*, 1999; Schroeter *et al.*, 2008), include plaque-free areas, sometimes with residual plaque cores, a moth-eaten appearance of remaining plaques, phagocytosed amyloid-β within microglia, and marked cerebral arteriolar and capillary amyloid angiopathy (Nicoll *et al.*, 2006), and are illustrated in Fig. 1. As expected, the single placebo case with neuropathologically confirmed Alzheimer’ disease (Case 15) had no evidence of plaque removal. In contrast, 14/16 (88%) of the patients with neuropathologically confirmed Alzheimer’s disease who had received the active vaccine had evidence of plaque removal: five had nearly complete plaque removal with only scattered small patches containing residual plaques, four had intermediate plaque removal (i.e. multiple and/or extensive plaque-free foci each involving a >1 cm length of cortical ribbon), five had very limited plaque removal (i.e. single and/or small plaque-free foci each involving a <1 cm length of cortical ribbon), whereas only two had no evidence of plaque removal.

#### Method 2

**Figure 2:**
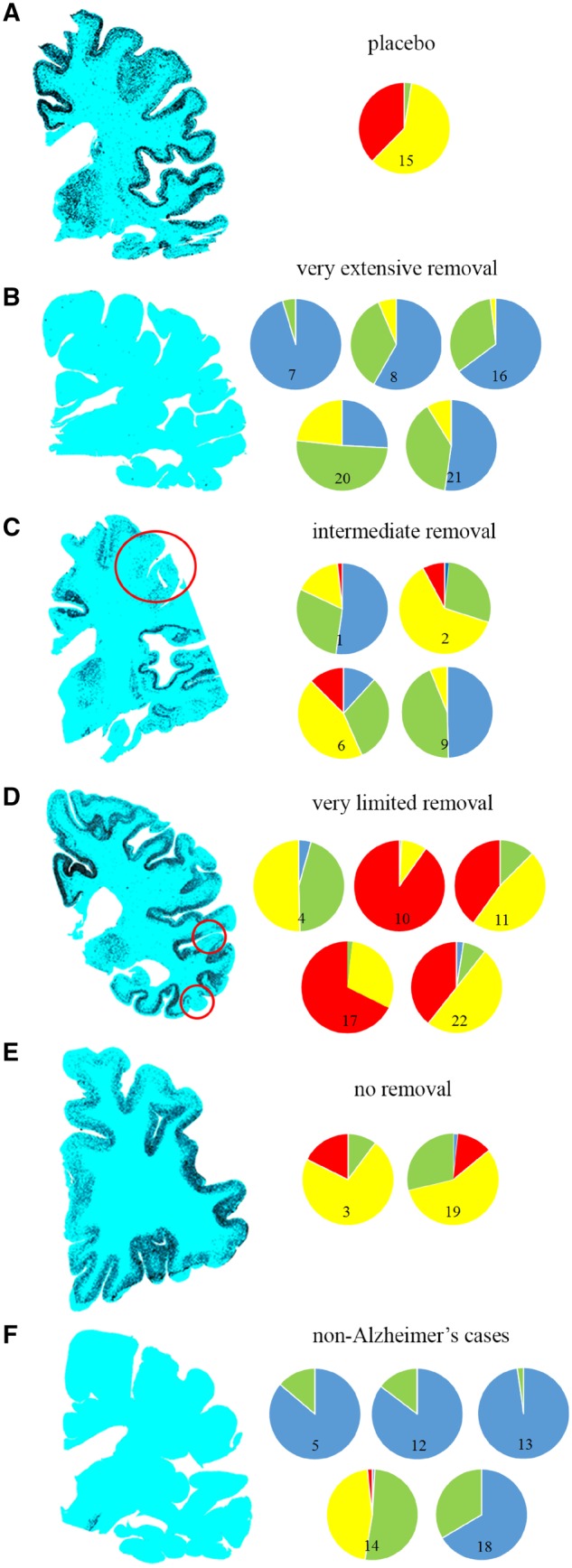
**Scans of coronal histological sections of cerebral hemisphere from participants in the AN1792 trial immunolabelled to demonstrate amyloid-β (appearing black).** The cases are grouped according to the extent of positive histological evidence of plaque removal on examination of all brain regions (Method 1). (**A**) Placebo with neuropathologically confirmed Alzheimer’s disease demonstrating a continuous band of plaques throughout the cerebral neocortex (Case 15). (**B**) Alzheimer’s subjects with nearly complete plaque removal (Cases 7, 8, 16, 20 and 21). (**C**) Alzheimer’s subjects with intermediate plaque removal (Cases 1, 2, 6 and 9). (**D**) Alzheimer’s subjects with limited plaque removal (Cases 4, 10, 11, 17 and 22). (**E**) Alzheimer’s subjects with no evidence of plaque removal (Cases 3 and 19). (**F**) Non-Alzheimer’s subjects (Cases 5, 12, 13, 14 and 18). Amyloid-β plaque density was scored throughout the neocortex of each hemisphere section using a CERAD-adapted method (Method 2) with the results colour-coded for illustrative purposes as: frequent plaques = red, moderate = yellow, sparse = green, none = blue, expressed as % of each category.

Quantification of amyloid-β plaques in coronal hemisphere sections (Fig. 2) showed that, in contrast to the single Alzheimer’s placebo case (Fig. 2A), the five cases assessed by Method 1 as having nearly complete removal of plaques all had >75% of the cortex scoring in the none/sparse plaque category (Fig. 2B). In contrast, the two immunized Alzheimer’s cases with no histological features of plaque removal (Fig. 2E) had >85% of the cortex containing moderate/frequent plaques. As expected, the plaque scores for the cases with neuropathologically diagnosed causes of dementia other than Alzheimer’s disease were predominantly none/sparse (Fig. 1F).

Among the immunized Alzheimer’s cases, no differences were observed for the amyloid-β plaque scores between the cerebrovascular territories (mean: anterior 1.59; middle 1.33; posterior 1.16; cerebral artery territories; *P = *0.485) or between the watershed compared to the non-watershed areas (mean watershed 1.37 versus non-watershed 1.46; *P = *0.793).

### Time course

Near-complete plaque removal was identified in the brains of two patients who died 14 years after immunization (Cases 20 and 21, 166 months and 173 months, respectively). Foci of plaque removal were present after as little as 4 months (Case 2). These findings indicate that plaque removal can occur rapidly and be sustained for many years after active amyloid-β immunization. The five patients with the most extensive plaque clearance had a mean survival of 115 months compared with 82 months for the remaining patients with Alzheimer’s disease (89 months excluding the participant who died from rupture of an abdominal aortic aneurysm 4 months after their first immunization). However, there was no significant relationship between the extent of plaque removal and survival time since first immunization dose (*r* = 0.182, *P* = 0.485), suggesting that extensive plaque removal does not confer survival benefit and that a prolonged time interval does not diminish the effect of the immunization. Interpretation of this analysis is hampered by the small numbers of subjects, wide age range of subjects at recruitment, and the fact that the cause of death of some subjects was due to co-incident disease rather than dementia (Table 1).

### Amyloid-β plaque scores and serum anti-AN1792 antibody titres

**Figure 3:**
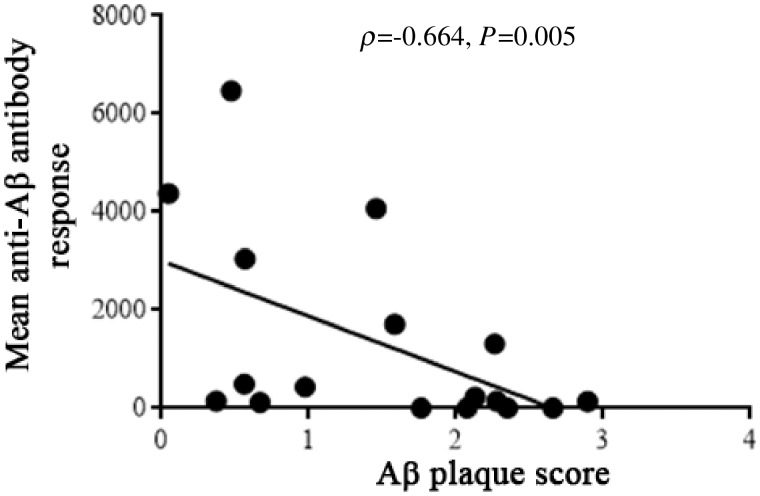
**Negative association between the mean anti-AN1792 antibody response during the trial period and post-mortem amyloid-β plaque score indicating that the magnitude of the immune response to AN1792 influenced the degree of amyloid-β removal from the brain.**.

Peripheral blood anti-AN1792 antibody titres available from the period of the trial (2000–2002) were analysed in relation to the amyloid-β plaque scores on post-mortem neuropathology (Table 2). In the neuropathologically confirmed Alzheimer’s cases, amyloid-β plaque scores showed a significant inverse correlation with both mean and peak anti-AN1792 antibody titres (ρ* = *−0.664, *P = *0.005; ρ = −0.617, *P = *0.011, respectively) (Fig. 3), despite the interval of several years in most cases between the antibody assays and the post-mortem assessments. Amyloid-β removal seemed to be most pronounced in the subjects who generated a substantial immune response to the vaccine, supporting the hypothesis that the anti-amyloid-β antibodies that are instrumental in removing the amyloid-β plaques from the brain.

### Tau

**Figure 4:**
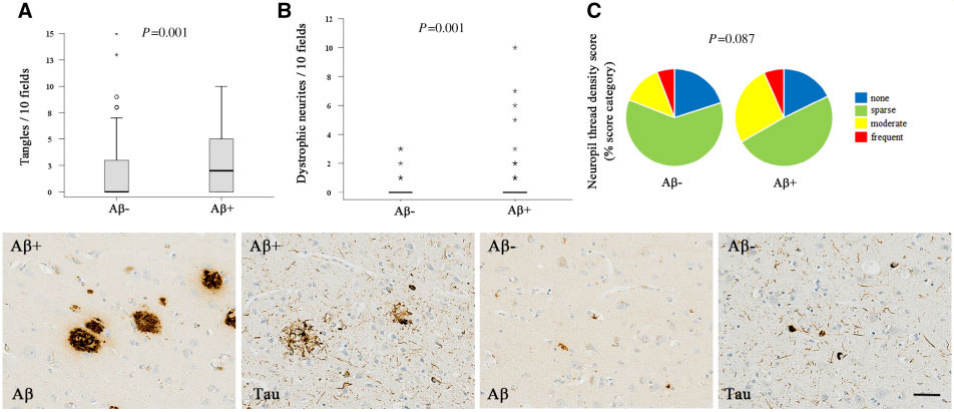
**The effect of amyloid-β removal was quantified for each of the different histological features of intraneuronal tau accumulation (tangles, dystrophic neurite clusters and neuropil threads) by comparing cortical regions with amyloid-β plaques remaining (Aβ+) to regions with amyloid-β plaques removed (Aβ−).** There were significantly fewer tangles and dystrophic neurite clusters in the regions where amyloid-β plaques had been removed in response to the amyloid-β immunotherapy; not significant for neuropil threads. Scale bar = 50 μm.

To determine the local effect on tau of removing amyloid-β from the cortex, we compared tau pathology between neocortical areas with remaining amyloid-β plaques (amyloid-β+) and those without remaining amyloid-β plaques (amyloid-β−) (Fig. 4). Tau-containing dystrophic neurite clusters and tau-containing neuronal cell bodies were significantly fewer in plaque-free regions (*P = *0.001), consistent with a local relative reduction in tau associated with amyloid-β immunization-induced removal of plaques. Nevertheless, 15/16 of the immunized Alzheimer’s subjects had progressed to Braak stage V/VI (Table 2) which reflects widespread anatomical distribution of aggregated tau within the brain, suggesting that spread of tau pathology continued despite the removal of plaques. The single immunized Alzheimer’s subject not reaching this advanced stage of tau distribution had died shortly after his first immunization dose at a relatively early stage of the disease (Case 2, Braak stage III/IV, 4 months). Of note, all five cases with nearly complete removal of plaques had an advanced stage of tangle distribution within the cerebrum (Braak stage V/VI).

### Meningoencephalitis

**Figure 5:**
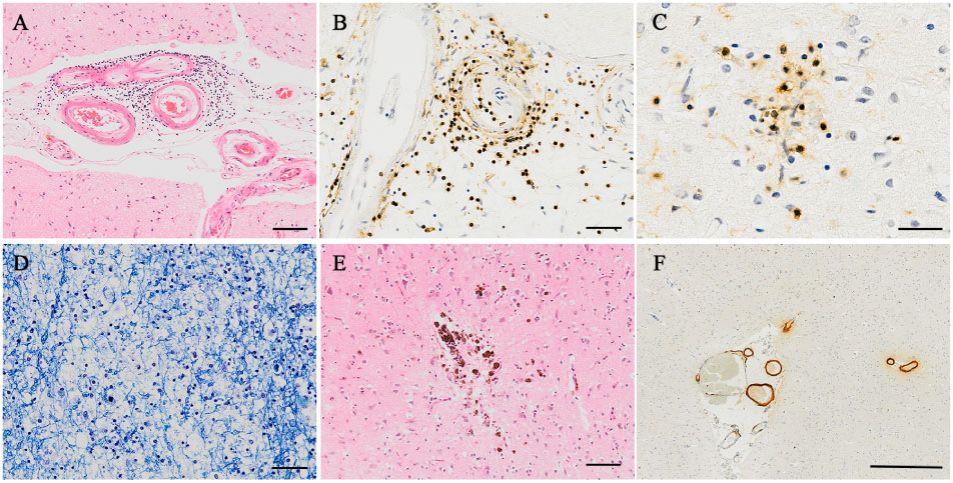
**Illustrations of the single case of post-immunization meningoencephalitis (Case 1).** Numerous lymphocytes in the subarachnoid space clustered around arteries showing the ‘double-barrel’ morphology associated with severe cerebral amyloid angiopathy (**A**) haematoxylin and eosin; and (**B**) CD3 immunohistochemistry for T lymphocytes. (**C**) Lymphocytes were relatively sparse in the cerebral cortex. (**D**) Cerebral white matter showed focal rarefaction of myelinated fibres (Kluver Barrera). (**E**) Old cortical microbleed indicated by the presence of a cluster of haemosiderin pigment granules. (**F**) Severe cerebral amyloid angiopathy with full thickness, full circumference involvement of the walls of leptomeningeal and parenchymal arteries by amyloid-β (immunohistochemistry for pan-amyloid-β). Scale bars = 100 μm in **A** and **E**; 50 μm in **B** and **D**; 30 μm in **C**; and 500 μm in **F**.

Only one of the 22 patients (case 1) was recognized to have had a clinical event corresponding to the post-immunization meningoencephalitis described in a subsequent multinational phase 2a trial of AN1792 (Orgogozo *et al.*, 2003). As part of a trial extension phase she had a fifth injection, with a reformulated preparation containing polysorbate-80 as used in the subsequent trial, and 6 weeks later she became unwell with dizzy spells, drowsiness, an unstable gait and fever. Brain imaging showed extensive alterations in the cerebral white matter bilaterally and enhancement on the brain surface. Although, the post-mortem analysis followed 11 months after the clinical episode, the neuropathological findings were striking with abundant leptomeningeal T lymphocytes, relatively scanty lymphocytes in the cerebral cortex, cortical microvascular lesions related to severe cerebral amyloid angiopathy, and oedema of the white matter (Nicoll *et al.*, 2003; illustrated in Fig. 5). Of note, 13/14 subjects with neuropathologically confirmed Alzheimer’s disease and histological evidence of plaque removal did not have such a clinical event and lacked a significant infiltrate of lymphocytes. Previous quantification showed in the meningoencephalitis case (Case 1), the presence of 1024.5 CD3+ T lymphocytes/100 fields in the leptomeninges and 65.5 in the grey matter compared with median values of 12 in leptomeninges and 1.8 in the grey matter in immunized Alzheimer cases without encephalitis and 14.2 in leptomeninges and 7.3 in the grey matter in non-immunized Alzheimer’s disease cases (Zotova *et al.*, 2013). This absence of a significant lymphocytic infiltrate in all but one of the AN1792-treated patients is consistent with the plaque removal activity following AN1792 immunization being primarily related to a humoral immune response.

### Cognitive function and cause of death

Although at the time of entry to the trial all participants satisfied the criteria for mild/moderate dementia, 19/22 were assessed as having severe dementia prior to death (Table 1), notably including all five patients with near complete clearance of plaques from the brain (Cases 7, 8, 16, 20 and 21). The late assessments were performed within a year of death so represent a good indication of the cognitive status in relation to the neuropathological findings. The remaining three participants had moderate dementia (Cases 2, 10 and 11), had a neuropathological diagnosis of Alzheimer’s disease and had received the active agent. One showed intermediate plaque removal, the other two had evidence of only very limited plaque clearance. One of these three participants had died from rupture of an abdominal aortic aneurysm just 4 months after first immunization. The other two died from bronchopneumonia, with dementia cited in the cause of death. A neurocognitive disorder was mentioned in the cause of death as stated on the death certificates for 19/22 participants. No patient died from a cause attributable to the immunization.

### Non-Alzheimer’s disease cases

Five of the 22 subjects (23%) had non-Alzheimer’s causes for dementia (Table 2). Consistent with their non-Alzheimer’s diagnoses, they predominantly had low plaque scores (Fig. 2F). Three of the non-Alzheimer’s subjects had received the active vaccine, only one of whom had evidence of circulating antibodies against AN1792 (Case 12).

## Discussion

Systematic post-mortem follow-up over a 15-year period of patients who had been enrolled in the first trial of amyloid-β immunization in Alzheimer’s disease has yielded unique insights into the effects of amyloid-β removal and the progression of disease. Such a long-term neuropathological analysis of a therapeutic trial for a neurological condition, in this case with a >25% post-mortem rate, has rarely, if ever, been performed previously. The study has limitations, including the single time point of analysis for each subject inevitable in a post-mortem study, in contrast with serial assessment of amyloid load now possible with *in vivo* PET imaging (Liu *et al.*, 2015). However, we were able to ascertain the long-term effects of active amyloid-β immunization on plaque clearance, analyse variation between cases, study the patchy nature of plaque removal, and exploit this patchy removal, by comparing areas with plaques removed to areas with plaques remaining in the same brains to act as internal controls, to study its effect on tau pathology. Therefore, the lack of significant numbers of placebo subjects coming to post-mortem neuropathological examination did not compromise these aims.

Here we report an important finding of the long-term effects of active amyloid-β immunization in not only removing plaques but keeping plaques at bay for up to 14 years, with the brains of some subjects rendered virtually plaque-free for many years after the therapeutic intervention had ceased. There are no published data comparable to this. Most clinical trials of amyloid-β immunotherapy for Alzheimer’s disease subsequent to AN1792 have used passive immunization strategies (Selkoe and Hardy, 2016). A particular advantage of active immunization, stimulating the host’s own immune system to produce the antibodies, over passive amyloid-β immunization in which exogenous antibodies are provided, is the prolonged effect achievable even in an elderly population, as demonstrated here. The importance of this observation is that if amyloid-β immunotherapy should prove successful in preventing Alzheimer’s disease, rather than treating established disease, then early active immunization would be an effective strategy, as we show here the level of the immune response is sufficient to stimulate and maintain the amyloid-β clearance over many years. Clinical trials of passive amyloid-β immunotherapy are currently in progress in groups at high risk of developing Alzheimer’s disease, including those with a familial autosomal dominant form of the disease due to *PSEN1* mutation or *APOE* ɛ4 homozygotes (Prevention and Generation studies) (The Lancet, 2012; National Institute on Aging, 2016). While these are important studies as a test of principle, widespread use in the population of passive immunotherapy over a period of many years or decades would probably be impracticable. In contrast, active immunization, analogous to immunization for the prevention of bacterial and viral infections, seems more feasible.

Another notable finding in this study is the post-mortem neuropathological observation that 5/22 participants (23%) had causes for their dementia other than Alzheimer’s disease. Trials without neuropathological follow-up lack definite knowledge of the diagnostic error, and inaccuracy of the clinical diagnosis of Alzheimer’s disease, have hampered the early immunotherapy trials. For example, consistent with our finding, *post hoc* analysis of a trial of the anti-amyloid-β antibody bapineuzumab showed that up to a third of subjects lacked amyloid on imaging, so probably did not have Alzheimer’s disease (Liu *et al.*, 2015). Clearly, the statistical power of trials of therapy for Alzheimer’s disease is severely compromised if a substantial proportion of the patients do not have the disease at which the potential therapy is targeted. More recent developments in trial design require at subject enrolment that brain amyloid is detected by PET scans and CSF analysis of amyloid-β and tau is consistent with Alzheimer’s disease. Both forms of assessment can be expected to have improved the accuracy of diagnosis in more recent trials (Selkoe and Hardy, 2016).

Most of the patients in our study showed a decline in cognitive function from mild/moderate dementia at trial entry to severe dementia prior to death. Of particular note, five patients with neuropathological confirmation of Alzheimer’s disease who had received the active vaccine, developed circulating anti-AN1792 antibodies and had nearly complete clearance of plaques, all progressed to severe dementia. Soluble/oligomeric amyloid-β also appeared to have been cleared in one such case examined previously (Maarouf *et al.*, 2010). This finding supports and strengthens our previous conclusion at 6 years of follow-up, although based at that stage on only two of the patients, that removing the plaques in established Alzheimer’s disease fails to halt the cognitive decline (Holmes *et al.*, 2008). This conclusion implies that in established Alzheimer’s disease, pathological components other than amyloid-β plaques—such as microglial activation and/or tau accumulation—predominate in perpetuating the progression of dementia.

Since the first amyloid-β immunotherapy trial (AN1792), the neuropathological follow-up of which is presented here, the landscape has changed considerably, with huge financial investment by the pharmaceutical industry and many thousands of patients with dementia exposed to various active and passive amyloid-β immunotherapies (Selkoe and Hardy, 2016). Studies to date have shown no or limited evidence of benefit to cognitive function. However, according to amyloid scan data, most trials have shown only modest effects on reducing amyloid plaque burden (Rinne *et al.*, 2010; Salloway *et al.*, 2014), contrasting with our neuropathological findings here relating to AN1792. A notable exception is the monoclonal antibody aducanumab (Sevigny *et al.*, 2016) of which a relatively small clinical trial of patients with a clinical diagnosis of prodromal or mild Alzheimer’s disease and visually positive amyloid-β PET scan using very high doses of antibody, yielded imaging evidence of very extensive amyloid clearance, comparable to that identified histologically in the current study. Stabilization of cognitive decline was demonstrated in this preliminary clinical trial with aducanumab in patients with evidence of amyloid removal on PET imaging.

Fourteen of 16 (88%) Alzheimer’s patients receiving the active agent in this study had evidence of plaque removal, but with considerable variability from case to case and striking patchiness of plaque removal in some cases. This is in contrast to the relatively uniform distribution of plaques throughout the cerebral neocortex typically seen in untreated Alzheimer’s disease. Similar patchiness of amyloid-β removal has been demonstrated with amyloid PET scans before and after treatment of patients in trials of subsequent immunotherapy agents (e.g. bapineuzumab) (Liu *et al.*, 2015). The National Institute on Aging-Alzheimer’s Association guidelines for neuropathological assessment of Alzheimer’s disease have been applied in this study to provide a standardized comparator with non-immunized Alzheimer’s cases (ABC, Table 2). This system is based on the progressive accumulation and anatomical progression of Alzheimer’s pathology which occurs during the natural history of the disease in the form of amyloid-β (A) and tau (B) and maximal focal density of neuritic plaques (C). This progressive accumulation of neuropathological features has been disrupted as a consequence of the amyloid-β immunization, and thus the ABC assessments under-represent the profound alterations in amyloid-β and tau pathology which are better reflected by the quantitative analyses shown. For example, Cases 7 and 8 had very low levels of plaque amyloid-β, in the cerebral cortex only, with none in the basal ganglia, brainstem or cerebellum (hence A1, as shown in Table 2), despite the advanced stage of distribution of tangles (B3, i.e. Braak V/VI), an overall pattern consistent with Alzheimer’s pathology in which most of the amyloid-β has been removed by the immunotherapy.

There are two likely mechanisms of removal of amyloid-β following immunization, with entry of antibodies into the brain and binding of anti-amyloid-β antibodies to plaques (Boche *et al.*, 2010*a*). First, opsonization as a consequence of antibody binding provokes microglia to phagocytose amyloid-β by binding of the IgG Fc region to Fc receptors on the microglial cell surface. Evidence for the occurrence of this mechanism in relation to AN1792-mediated plaque removal comes from the presence of amyloid-β within the cytoplasm of microglia as we have demonstrated previously by double label confocal microscopy (Nicoll *et al.*, 2006; Boche *et al.*, 2010*a*) giving a characteristic appearance of small dense clusters of amyloid-β immunoreactivity, often amongst ‘moth-eaten’ or disrupted plaques as illustrated in Fig. 1C. This appears to be followed, once plaques have been removed from a region of cortex, by subsequent dispersal (Zotova *et al.*, 2011) and downregulation of microglia (Zotova *et al.*, 2013). The second mechanism of plaque removal appears to involve solubilization of amyloid-β, with high levels of soluble amyloid-β detected in some immunized cases (Maarouf *et al.*, 2010). Solubilization seems to result in translocation of amyloid-β to the walls of arteries and capillaries resulting in an increased severity of cerebral amyloid angiopathy (Boche *et al.*, 2008; Sakai *et al.*, 2014), a feature that may be localized and transient. Although cerebral amyloid angiopathy cannot yet be readily imaged *in vivo* in the human brain, evidence suggests that it is a major contributor to the complication of amyloid-related imaging abnormalities (ARIA) hampering more recent and current clinical trials, including those of passive immunotherapy (Sperling *et al.*, 2011; Sevigny *et al.*, 2016) as discussed below.

Only 1 of 22 patients (Case 1) in our study of the phase 1 trial of AN1792 was observed to have the side effect termed ‘meningoencephalitis’ described in the subsequent phase 2a trial (Orgogozo *et al.*, 2003), which halted further development with AN1792 (Vellas *et al.*, 2009). Interestingly, the complication in Case 1 developed only during a trial extension phase shortly after the patient had a fifth injection, with a reformulated preparation containing the solubilizing agent polysorbate-80 as used in the subsequent phase 2a trial. There is little information on the pathological substrate of this complication, limited to two patients who received AN1792, including the initial description of Case 1 in this present study (Nicoll *et al.*, 2003) and one subsequent case from the phase 2a trial (Ferrer *et al.*, 2004). However, the pathology shares features with sporadic amyloid-β-related angiitis, in which histological evidence has been observed of a T lymphocyte immune response to amyloid-β in the cerebral vasculature and also, in some cases, in the parenchyma (Scolding *et al.*, 2005). We suggested previously that the complications experienced with AN1792 were a consequence of solubilization of plaques with translocation of amyloid-β to the vasculature, increasing the severity of cerebral amyloid angiopathy and consequently generating microhaemorrhages and white matter oedema (Boche *et al.*, 2008, 2010*a*). It was unclear whether or not a lymphocytic reaction was essential to the development of this complication and we predicted that it may be encountered in any therapy designed to remove amyloid-β from the brain. Indeed, a recurring side effect in amyloid-β immunotherapy trials subsequent to AN1792 has been the so-called ‘ARIA’ (Sperling *et al.*, 2011), mainly in the form of focal white matter oedema and cortical microhaemorrhages, occurring in up to half of treated patients (Sevigny *et al.*, 2016) and limiting antibody dosage (Sperling *et al.*, 2011). ARIA is identified on MRI scans and is usually, but not always, asymptomatic. In retrospect, the imaging features of the meningoencephalitis experienced with AN1792 are similar to ARIA; however, pathological information on ARIA, for comparison with the findings from AN1792, is lacking.

CSF and blood samples from these patients were not available to permit assessment of a time period of plaque-binding antibodies following immunization. However, anti-AN1792 antibodies were assayed in a subgroup of participants in a previous study (Holmes *et al.*, 2008) at a time point 6 years after immunization with AN1792 and showed persistence of circulating anti- AN1792 antibodies at that stage. We explored the presence of antibodies on remaining plaques previously when we had 11/16 of the immunized Alzheimer cases presented in the current study (Zotova *et al.*, 2013). We found IgG to be variably present on plaques both in immunized and non-immunized Alzheimer’s disease cases, with no consistent pattern. We were not able to distinguish between plaque-bound antibodies specifically directed against amyloid-β, whether generated in response to the AN1792 active vaccine or not, from antibodies bound to other plaque components due to technological limitations. However, demonstration of IgG bound to plaques does confirm the ability of IgG to cross the blood–brain barrier. As evidence suggests that plaque removal requires access of antibodies from the bloodstream into the brain parenchyma, this raises the possibility that vascular factors are a determinant in the success of plaque removal. We explored whether the patchiness of plaque removal could reflect anatomical cerebral arterial territories or arterial watershed regions but found that the cerebrovascular anatomy did not explain the heterogeneity in plaque removal. This patchiness of removal has been a recurring phenomenon in subsequent amyloid-β immunotherapy trials (Sperling *et al.*, 2012; Sevigny *et al.*, 2016) and remains unexplained, but could perhaps be due to local alterations in the blood–brain barrier associated with an ageing cerebral vasculature, damage to pericytes (Sagare *et al.*, 2013; Miners *et al.*, 2018), or specific features of Alzheimer’s pathology such as cerebral amyloid angiopathy (Skrobot *et al.*, 2016).

We took advantage of this patchy nature of plaque removal to investigate the effects on tau, comparing regions of cortex from which plaques had been removed with immediately adjacent regions where plaques remained. Our findings demonstrate that plaque removal mediated by active amyloid-β immunization can influence tau accumulation for up to 14 years, reducing local accumulation of tangles and dystrophic neurites within the cerebral cortex. This implies that late in the disease, in the absence of immunotherapy, amyloid-β continues to drive local accumulation of tau.

The reduction in tau-containing plaque-associated dystrophic neurites is a striking consequence of immunization-mediated removal of amyloid-β from the cortex and has been described previously (Nicoll *et al.*, 2003; Ferrer *et al.*, 2004; Boche *et al.*, 2010*b*). The mechanism for this localized reduction in tau is unclear, but it could conceivably reflect, firstly, restored neuronal homeostasis leading to dismantling of the aggregated tau within the neuronal processes. Previous studies have identified a reduction in neuritic curvature which would be consistent with ‘straightening’ of abnormal neurites after amyloid-β removal (Serrano-Pozo *et al.*, 2010; Paquet *et al.*, 2015) and observation after immunization of a reduction in tau kinases responsible for phosphorylating tau, would support this possibility (Ferrer *et al.*, 2004; Amin *et al.*, 2015). No extra-neuronal tau was identified and residual tau-negative dystrophic neurites were not identified. Further support for improved ‘neuronal health’ after removal of amyloid-β would include downregulated apoptosis and autophagy (Paquet *et al.*, 2018). In addition, a reduction of local synaptic abnormalities was suggested by one case study (Ferrer *et al.*, 2004); however, overall cortical synaptic status assessed by synaptophysin immunohistochemistry did not support this finding (Boche *et al.*, 2010*a*). A second possible explanation for the reduction in tau-containing dystrophic neurites is the removal of the dystrophic neurites by microglial phagocytosis. Identification of a reduced neuropil density after immunization could support this mechanism (Paquet *et al.*, 2015); however, phagocytosed tau, unlike amyloid-β, was not detected in microglia in areas of cortex cleared of plaques making this latter possibility less likely. It is notable that all components of the plaque appear to be disassembled following removal of amyloid-β by immunotherapy, including: tau within the dystrophic neurites, the dystrophic neurites themselves, and also microglia (Zotova *et al.*, 2011), astrocytes and APOE associated with plaques (Nicoll *et al.*, 2006). The neuritic plaque is the single point at which many of the key features of Alzheimer’s disease pathophysiology converge: amyloid-β, tau, microglia and APOE and therefore it seems likely to represent a key component of the evolution of the disease process.

Nevertheless, we found that all patients had progressed to Braak stage V/VI, which represents an advanced spread of tangle pathology throughout the brain. Tau has been proposed to spread through the brain from neuron to neuron by a ‘prion-like’ mechanism (Clavaguera *et al.*, 2009; Spillantini and Goedert, 2013). Our observations suggest that removal of plaque-associated amyloid-β after the initiation of tau accumulation may not prevent the continued spread of tau pathology, a contention supported by our previous observations when compared to a non-immunized Alzheimer’s cohort (Boche *et al.*, 2010*b*). This finding is relevant to the lack of observed benefit to cognitive function in our study and subsequent phase III trials of amyloid-β immunotherapy in established Alzheimer’s disease, as cognitive dysfunction is known to correlate more closely with accumulation of tau than that of amyloid-β (Minett *et al.*, 2016). Tau ligands for PET imaging are becoming available and may in future provide further information on the persistence or progression of tau pathology after amyloid-β immunotherapy. There is growing interest in use of amyloid-β immunotherapy early in the course of the Alzheimer’s process, for example at the stage of mild cognitive impairment, or even as a preventative measure (The Lancet, 2012). Our findings in this study in relation to tau suggest that very early intervention, before significant self-propagating accumulation of tau, is desirable to prevent progression. The outcome of such preventative studies, the ultimate test for the amyloid cascade hypothesis, is eagerly awaited.

Amyloid-β immunotherapy trials subsequent to AN1792 have represented a huge financial investment amounting to many hundreds of millions of dollars and it is disappointing that no systematic post-mortem neuropathology follow-up seems to have been organized for those trials to help understand the reasons for the limited clinical benefit and to inform future research studies. Even though the trials of amyloid-β immunization have so far failed to meet their primary cognitive end-points, analysis of the varying neuropathological effects of targeting different specific forms of amyloid-β, such as oligomeric and fibrillary forms, could provide important information on key correlates between Alzheimer pathophysiology, the immune response and cognitive function that are as yet poorly understood.
